# Pregnancy glycaemia and cord-blood levels of insulin and leptin in Pakistani and white British mother–offspring pairs: findings from a prospective pregnancy cohort

**DOI:** 10.1007/s00125-014-3386-6

**Published:** 2014-10-03

**Authors:** Debbie A. Lawlor, Jane West, Lesley Fairley, Scott M. Nelson, Raj S. Bhopal, Derek Tuffnell, Dilys J. Freeman, John Wright, Donald C. Whitelaw, Naveed Sattar

**Affiliations:** 1 MRC Integrative Epidemiology Unit at the University of Bristol, Oakfield House, Oakfield Grove, Bristol, BS8 2BN UK; 2 School of Social and Community Medicine, University of Bristol, Bristol, UK; 3 Bradford Institute for Health Research, Bradford Royal Infirmary, Bradford, UK; 4 School of Medicine, University of Glasgow, Glasgow, UK; 5 Edinburgh Ethnicity and Health Research Group, Centre for Population Health Sciences, University of Edinburgh, Edinburgh, UK; 6 Women’s and Newborn Unit, Bradford Royal Infirmary, Bradford, UK; 7 Institute of Cardiovascular and Medical Sciences, University of Glasgow, Glasgow, UK; 8 Department of Diabetes and Endocrinology, Bradford Royal Infirmary, Bradford, UK

**Keywords:** Birthweight, Cord-blood insulin, Cord-blood leptin, Epidemiology, Ethnicity, Gestational diabetes, Glucose

## Abstract

**Aims/hypothesis:**

To determine the extent to which gestational fasting and postload levels of glucose explain differences in infant fat mass between UK-born Pakistani and white British infants.

**Methods:**

Analyses were undertaken in a prospective pregnancy cohort study of 1,415 women and their singleton live-born infants (629 white British and 786 Pakistani). Infant fat mass was assessed by cord-blood leptin levels and fetal insulin secretion by cord-blood insulin levels. Maternal OGTTs were completed at 26–28 weeks of gestation.

**Results:**

Pakistani women had higher fasting and postload glucose levels and greater incidence of gestational diabetes than white British women. Higher fasting and postload glucose levels were associated with higher cord-blood levels of insulin and leptin in all participants, irrespective of ethnicity. Cord-blood leptin levels were 16% (95% CI 6, 26) higher in Pakistani than in white British infants. After adjustment for fasting glucose levels, this difference attenuated to 7% (−3, 16), and with additional adjustment for cord-blood insulin levels it attenuated further to 5% (−4, 14). Path analyses supported the hypothesis that fasting glucose levels mediate the relationship of Pakistani ethnicity to greater fat mass at birth, as measured by cord-blood leptin levels; on average, 19% of this mediation involved fetal insulin secretion. Postload glucose levels did not act as an important mediator of ethnic differences in cord-blood leptin levels. Results were very similar when 130 women with gestational diabetes were removed.

**Conclusions/interpretation:**

These novel findings suggest a role of maternal pregnancy glycaemia in mediating differences in fat mass between Pakistani and white British infants.

**Electronic supplementary material:**

The online version of this article (doi:10.1007/s00125-014-3386-6) contains peer-reviewed but unedited supplementary material, which is available to authorised users.

## Introduction

For a given BMI, South Asian compared with white European adults have greater fat mass, leading to the suggestion that they have a ‘thin-fat’ insulin-resistant phenotype that underlies their increased type 2 diabetes risk [1–3]. Recent evidence suggests that this phenotype is present in children [4–6], and possibly at birth [7]. For a given birthweight, South Asian babies born in India have been found to be fatter (as measured by subscapular and triceps skinfolds or cord-blood leptin levels) than white European babies born in Europe or the USA [7–9]. These differences may reflect different socioeconomic, lifestyle and healthcare characteristics between people living in India compared with those in Western countries. However, we have recently shown similar results when comparing babies of Pakistani origin conceived and born in the UK with white British babies born in the same maternity unit [10].

Greater gestational hyperglycaemia amongst South Asian women [11–14] is a plausible exposure that could contribute to greater fat mass at birth in South Asian infants because (1) greater circulating pregnancy fasting and postload glucose concentrations promote fetal insulin secretion and fat accretion in a linear dose–response fashion [15–19]; and (2) an increased risk of gestational diabetes mellitus (GDM), hyperglycaemia and type 2 diabetes in people of South Asian origin is well established [1, 11–14, 20–25].

The aim of this study was to determine the extent to which differences between Pakistani and white British women in gestational glucose levels mediate differences in cord-blood leptin levels (a valid proxy for birth fat mass that has been used in previous studies for this purpose [7, 10, 26, 27]). We also investigated the role of cord-blood insulin levels in these relationships. This work extends our earlier work [10], which did not explore possible mediators of ethnic differences in cord-blood leptin levels at birth. To the best of our knowledge, the role of maternal gestational glycaemia in mediating differences in infant fatness between Pakistani (or any other South Asian group) and white British infants has not been previously explored.

## Methods

This study was approved by the Bradford Research Ethics Committee (ref 06/Q1202/48). All participants provided written informed consent. The study was undertaken in a planned pseudo-randomly selected subgroup of the Born in Bradford (BiB) cohort in whom relevant cord-blood outcomes were assayed. BiB is a prospective pregnancy cohort study that collected detailed information from 12,453 women who experienced 13,776 pregnancies resulting in 13,818 births [28]. To be eligible for BiB, women had to be booked to give birth between March 2007 and December 2010 in the maternity department at Bradford Royal Infirmary, in the North of England [28].

BiB women were recruited primarily at their OGTT appointment (26–28 weeks’ gestation). All women booked for delivery in Bradford are offered a 75 g OGTT (comprising fasting and 120 min postload samples) at around 26–28 weeks’ gestation. At recruitment, BiB participants completed an interviewer-administered questionnaire. Interviews were conducted in English or in a range of South Asian languages, including Urdu, Mirpuri, Bengali and Punjabi [28].

**Fig. 1:**
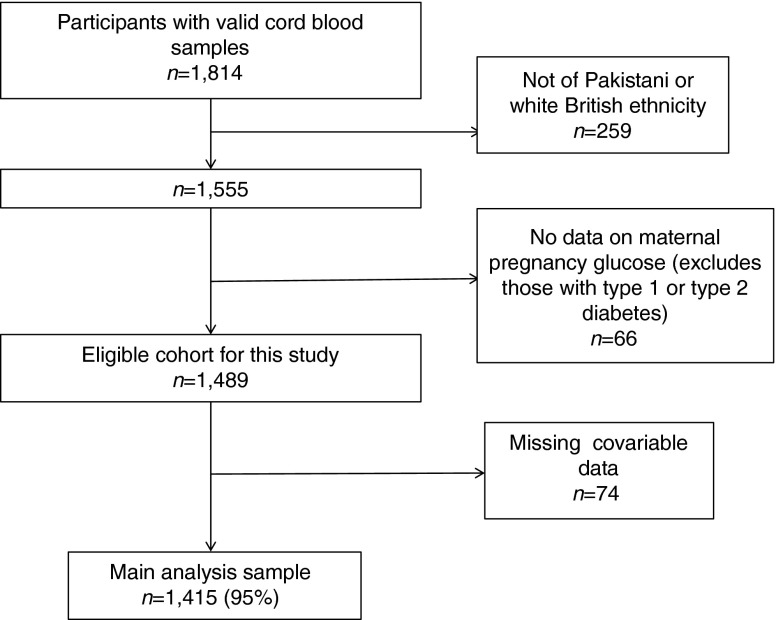
Flow diagram of participants

To be eligible for the cord-blood substudy, BiB participants had to have a live birth from a singleton pregnancy that occurred between mid-October 2008 and mid-October 2009. Electronic supplementary material (ESM) Table 1 shows that the distributions of participant characteristics are similar in the subgroup used for this study and those in the whole cohort of singleton live births. Figure 1 shows further exclusions from this study. Participants had to be of Pakistani or white British origin. The BiB cohort includes all eligible women, irrespective of their ethnic origin, but for this study other ethnic groups were either too small to be examined separately or were not relevant to the comparison of South Asian and white European ethnicity. Participants who had not completed an OGTT were excluded. Over 80% of the whole BiB cohort who were eligible for an OGTT completed it and had valid data on fasting and postload glucose levels; their characteristics were similar to those of participants who did not complete the OGTT [28]. Any women with pre-existing diabetes were not invited to have an OGTT, thus by excluding women without OGTT results we were also excluding any women with diagnosed existing diabetes. Following these exclusions, there were 1,489 eligible participants for this study and, of these, 1,415 (95%) had complete data on all covariables included in any model; these 1415 form the main analysis sample for this study (Fig. 1).

To be defined as Pakistani, women had to state that they belonged to category 10 of the UK Office of National Statistics criteria (Asian/British Asian subcategory and labelled Pakistani) [29]. To be defined as white British, women had to state that they belonged to category 1 (white subcategory and labelled English/Welsh/Scottish/Northern Irish/British). Full details of all of the Office of National Statistics categories are provided in the ESM Methods. For Pakistani women, additional information on their country of birth and that of their partner and their parents was also collected. This confirmed South Asian (Indian, Pakistani or Bangladeshi) familial origin for all who had reported that they were Pakistani. In a previous publication we have shown that differences in cord-blood leptin levels between Pakistani and white British infants were the same irrespective of place of birth of parents and grandparents (i.e. generation of migration of the Pakistani women) [10], and have not explored that further in this paper.

Participants completed a morning OGTT after fasting overnight. Fasting blood samples were taken for immediate analysis. Participants then consumed a standard solution containing the equivalent of 75 g anhydrous glucose over 5 min. After 120 min a second plasma sample was taken. All glucose assays were completed in the same laboratory in Bradford, using a glucose oxidase method, with laboratory staff blinded to the participants’ ethnicity or other characteristics. GDM was defined according to modified WHO criteria operating at the time these women were pregnant [30], as either fasting glucose ≥6.1 mmol/l or 2 h glucose ≥7.8 mmol/l. The modification that was applied in Bradford was using a fasting glucose threshold of ≥6.1 mmol/l. Although at the time the threshold recommended by WHO was ≥7.0 mmol/l, GDM was defined, including by the WHO, as any impaired glucose tolerance diagnosed for the first time in pregnancy, hence we used the threshold indicative of impaired fasting glucose. As a consequence, the definition being used in these women is similar to the recently proposed International Association of Diabetes and Pregnancy Study Group thresholds, which are included in the new WHO guidance [31].

Venous cord-blood samples were obtained at delivery by the attending midwife, following research protocols. Samples were refrigerated at 4°C in EDTA tubes until collected by laboratory staff within 12 h. Samples were then spun, frozen and stored at −80°C. Insulin is known to be stable in EDTA samples at room temperature for over 24 h [32]. The samples were transferred to Glasgow for completion of leptin and insulin assays. Leptin was measured by a highly sensitive in-house ELISA validated against the commercially available Linco assay, as detailed previously [33]. Insulin was measured by an ultrasensitive solid-phase two-site immunoassay ELISA (Mercodia, Uppsala, Sweden) that does not cross-react with proinsulin. Laboratory staff were blinded to the participants’ ethnicity and other characteristics.

Maternal age, education and smoking data were obtained from the interviewer-administered questionnaire completed at recruitment. Maternal education was equivalised to UK standard attainments and participants were included in one of five mutually exclusive categories (full details in the ESM Methods). Maternal smoking was categorised as never, past (but not during this index pregnancy), current/during the index pregnancy. Maternal BMI was calculated from height measured at the time of recruitment and from weight measured at first antenatal clinic, which was abstracted from the antenatal medical records; median (interquartile range) gestational age of the mothers at this booking clinic was 12 (11–14) weeks. Maternal parity, gestational age (to the last completed week) and infant birthweight and sex were abstracted from the antenatal/obstetric medical records.

### Statistical analyses

Maternal glucose levels and cord-blood levels of insulin and leptin were positively skewed and were described using medians and interquartile range. In regression analyses that used these variables as exposures or mediators (i.e. independent variables) they were used in their original form (mmol/l for glucose and pmol/l for cord-blood insulin) and in those that used them as outcomes they were natural log transformed so that the residuals in all models were approximately normally distributed. The resultant regression coefficients were transformed so that they represent differences in means as a percentage (%) per unit of exposure.

We tested our hypotheses in two ways. First, by a series of separate linear regression models that examined the associations of (1) ethnicity with fasting and postload glucose levels; (2) gestational fasting and postload glucose levels with cord-blood levels of insulin and leptin; (3) cord-blood insulin levels with cord-blood leptin levels; and (4) ethnicity with cord-blood levels of insulin and leptin. In these regression models we adjusted for maternal age, parity, smoking, education and BMI, gestational age and offspring sex. Details of the rationale for adjusting for these covariables are provided in the ESM Methods. In regression analyses for the association between ethnicity and cord-blood leptin levels, we additionally adjusted for potential mediation by fasting/postload glucose levels and cord-blood insulin levels.

Second, we used path analysis to simultaneously run all of the equations from the different regression analyses and pictorially show the standardised regression coefficients between exposures, potential mediators and outcomes. We included all of the same covariables in this path analysis as in the serial regression analysis, although on the figures we show only the results for the main exposure, outcome and mediating pathways. The path analyses were done with all variables standardised to have mean 0 and standard deviation 1 and run using the sem command in Stata.

We examined whether associations of glucose with cord-blood levels of insulin and leptin were linear in each ethnic group using fractional polynomials [34]. Differences between white British and Pakistani participants in the associations between gestational fasting and postload glucose levels with outcomes were tested using an interaction (ethnicity*glucose) test.

The main association analyses were completed in all 1,415 mother–offspring pairs and also in the 1,285 pairs after removal of the 130 pairs in whom the mother met the criteria for GDM.

## Results

ESM Table 2 shows characteristics for all participants and separately for Pakistani and white British women. Pakistani women were on average older, had greater parity, were much less likely to smoke and were more likely to be in the lowest and highest educational attainment categories. They had higher gestational fasting and postload glucose levels and lower BMI. Pakistani infants had higher cord-blood levels of insulin and leptin and lower birthweight than white British infants. None of the associations examined in this study differed by offspring sex (all *p*_interaction_ ≥ 0.2) and all regression analyses are presented with both sexes combined.

Cord-blood leptin level was positively associated with birthweight in both ethnic groups (ESM Fig. 1). For a 1 unit increase in logged cord-blood leptin level, birthweight increased by 311 g (95% CI 267, 355) in white British women and 263 g (95% CI 227, 302) in Pakistani women (*p*_interaction_ = 0.11). *R* ^2^ values indicated that cord-blood leptin levels explained 25% and 22% of the variation in birthweight in white British and Pakistani infants, respectively.

One hundred and thirty (9%) of the women were identified as having GDM; it was more common in Pakistani (*n* = 97 [12%]) than white British (*n* = 33 [5%]) women (*p* < 0.001). For the vast majority of women who met the criteria, GDM was diagnosed because of a high postload glucose level, with 77% of Pakistani and 88% of white British women with GDM having a postload glucose level of ≥ 7.8 mmol/l but a fasting glucose level in the normal range.

**Fig. 2:**
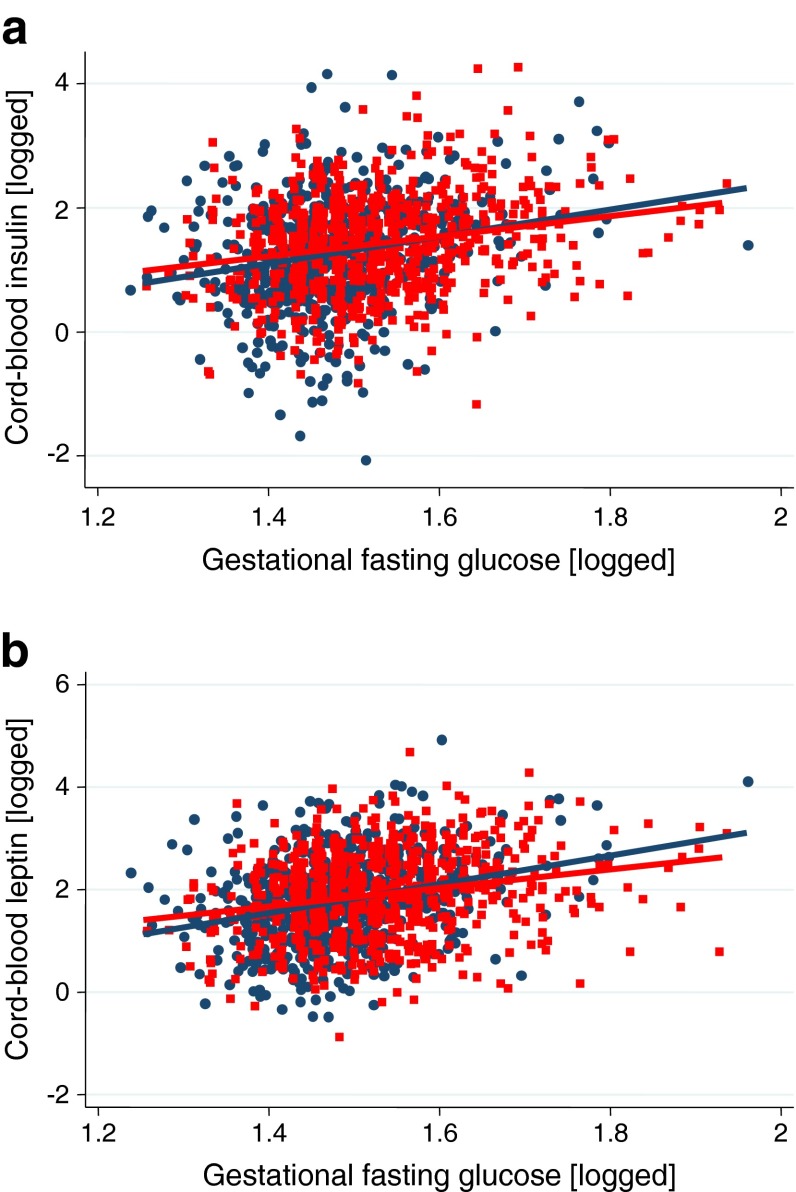
Association of maternal gestational fasting glucose levels with infant cord-blood levels of insulin (**a**) and leptin (**b**) in white British (blue circles; *n* = 629) and Pakistani (red squares; *n* = 786) mother–infant pairs

Figure 2 shows the scatter plots and unadjusted predicted regression lines for the associations of fasting glucose with levels of cord-blood insulin (Fig. 2a) and cord-blood leptin (Fig. 2b) in all participants. ESM Fig. 2 shows similar results for postload glucose and ESM Fig. 3 indicates the results when women with GDM are removed. In both Pakistani and white British participants, and irrespective of whether women who met criteria for GDM were removed or not, there were linear increases in levels of cord-blood leptin and cord-blood insulin across the distribution of increasing maternal gestational levels of fasting and postload glucose. The fractional polynomial models supported linear associations of both fasting and postload glucose levels with cord-blood levels of insulin and leptin in both Pakistani and white British women (all *p* values ≤0.001 for linear association and >0.2 for any power terms).

The linear positive associations of maternal gestational levels of fasting and postload glucose with cord-blood levels of insulin and leptin remained with adjustment for confounders in both the whole cohort (ESM Table 3) and in those who did not meet criteria for GDM (ESM Table 4). For cord-blood levels of insulin and leptin, the magnitudes of association were greater with fasting than with postload glucose levels (Table 1). Associations of fasting glucose levels with cord-blood levels of leptin and insulin were somewhat stronger when women with GDM were removed from the analyses than when all participants were included in analyses (Table 1). After adjustment for confounding factors, the magnitudes of association of fasting and postload glucose levels with cord-blood levels of insulin and leptin were similar in the two ethnic groups. Cord-blood insulin level was positively associated with cord-blood leptin level in both ethnic groups, again with similar magnitudes of association between the two ethnic groups (Table 1).

**Table 1:** Confounder-adjusted associations of gestational fasting and postload glucose levels with cord-blood levels of insulin and leptin in white British and Pakistani women in all participants (*n* = 1415) and in participants without GDM (*n* = 1285)

Outcome		Mean difference in outcome for each maternal exposure (95% CI)		*p*_interaction_^a^
		White British *n* = 629^b^ *n* = 596^c^	Pakistani *n* = 786^b^ *n* = 689^c^	
Exposure = fasting glucose level (per 1 mmol/l)				
Cord-blood insulin level (%)	All participants	30.2 (15.6, 44.8)	24.0 (15.4, 32.5)	0.43
	Participants without GDM	40.0 (21.6, 58.2)	24.7 (10.6, 38.8)	0.14
Cord-blood leptin level (%)	All participants	42.2 (29.0, 55.4)	29.4 (20.7, 38.0)	0.10
	Participants without GDM	48.9 (32.2, 65.6)	37.0 (22.7, 51.3)	0.24
Exposure = postload (120 min) glucose level (per 1 mmol/l)				
Cord-blood insulin level (%)	All participants	6.9 (1.9, 11.2)	7.5 (4.5, 10.5)	0.91
	Participants without GDM	7.5 (1.3, 13.8)	5.0 (0.0, 10.5)	0.43
Cord-blood leptin level (%)	All participants	8.2 (3.6, 12.8)	7.8 (4.8, 10.9)	0.79
	Participants without GDM	11.1 (5.4, 16.8)	8.6 (3.1, 14.1)	0.44
Exposure = cord-blood insulin level (per pmol/l)				
Cord-blood leptin level (%)	All participants	0.6 (0.4, 0.7)	0.6 (0.5, 0.7)	0.55
	Participants without GDM	0.7 (0.5, 0.8)	0.7 (0.5, 0.8)	0.80

All results are mean differences in the outcome expressed on a percentage scale and are adjusted for potential confounders: maternal age, BMI, parity, smoking, education, gestational age and offspring sex. The null value is 0 for all results

^a^Testing the null hypothesis that associations differ between white British and Pakistani pairs

^b^All participants

^c^Participants without GDM

Higher cord-blood insulin levels in Pakistani compared with white British infants remained after adjustment for confounders in both the whole cohort and when women with GDM were removed (Table 2). Adjustment for potential mediation by maternal fasting glucose levels attenuated the confounder-adjusted difference completely to the null in the whole cohort and markedly when those with GDM were excluded, whereas adjustment for postload glucose levels reduced the difference only slightly.

**Table 2:** Multivariable analyses of differences in cord-blood levels of leptin and insulin between Pakistani and white British mother–offspring pairs in all participants and in participants without GDM

Outcome	Model^a^	Mean difference (%) in outcome comparing Pakistani to white British (95% CI)	
		All participants *n* = 1,415	Participants without GDM *n* = 1,285
Cord-blood insulin level (%)	Model 1: unadjusted	14 (6, 22)	10 (1, 18)
	Model 2: confounder	9 (0, 19)	9 (0.4, 18)
	Model 3: confounder and gestational fasting glucose level	0 (−9, 11)	5 (−4, 14)
	Model 4: confounder and gestational postload glucose level	5 (−5, 15)	8 (−1, 17)
	Model 5: confounder and gestational fasting and postload glucose levels	0 (−10, 10)	4 (−5, 13)
Cord-blood leptin level (%)	Model 1: unadjusted	13 (4, 21)	9 (0.2, 19)
	Model 2: confounder	16 (6, 26)	12 (4, 21)
	Model 3: confounder and gestational fasting glucose level	7 (−3, 16)	6 (−3, 14)
	Model 4: confounder and gestational postload glucose level	12 (3, 22)	10 (1, 19)
	Model 5: confounder and gestational fasting and postload glucose levels	6 (−4, 16)	5 (−4, 14)
	Model 6: confounder and gestational fasting glucose and cord-blood insulin levels	5 (−4, 14)	4 (−4, 12)

All results are mean differences of outcome comparing Pakistani mother–offspring pairs to White British mother–offspring pairs (reference group); positive values indicate higher levels of outcomes in Pakistani pairs and the null value for all results is 0

^a^Model 1, no adjustment; model 2, adjustment for potential confounding by maternal age, BMI, parity, smoking, education, gestational age and offspring sex; model 3, as model 2 plus adjustment for gestational fasting glucose level; model 4, as model 2 plus adjustment for gestational postload glucose level; model 5, as model 2 plus adjustment for gestational fasting and postload glucose levels; model 6, as model 2 plus adjustment for gestational fasting glucose and cord-blood insulin levels

Cord-blood leptin levels remained higher in Pakistani than white British infants after adjustment for confounders, being 16% (95% CI 6, 26) and 12% (95% CI 4, 21) higher in the whole cohort and the cohort without GDM, respectively. In both cohorts, adjustment for potential mediation by fasting glucose levels reduced the ethnic difference by approximately 50% and statistically the difference was consistent with the null, whereas adjustment for postload glucose levels resulted in very little mediation. With additional adjustment (for confounders and mediation by fasting glucose levels and cord-blood insulin levels), there was a further attenuation to the null of the ethnic difference in cord-blood leptin levels (Table 2).

**Fig. 3:**
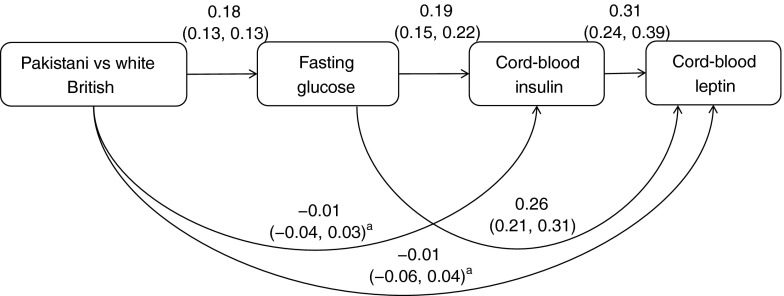
Path analysis for differences in cord-blood leptin levels between Pakistani and white British individuals (*n* = 1415). The results are standardised regression coefficients, with their 95% CIs, and are adjusted for maternal age, parity and education, infant sex and gestational age. They are interpreted as the adjusted change in outcome (box at the end of the arrow head) in SD units per category (ethnicity) or per SD of the exposure (arrow start). *p* < 0.001 for all results except those indicated ^a^ *p* ≥ 0.50

Figure 3 shows the results of the path analyses for the ethnic difference in cord-blood leptin levels in the whole cohort (with equivalent results when those with GDM were removed shown in ESM Fig. 4). The confounder-adjusted partial correlation coefficient for the difference in cord-blood leptin levels between Pakistani and white British women was 0.058 (*p* < 0.001), i.e. Pakistani infants had levels of cord-blood leptin that were 0.058 of an SD higher than white British infants. This difference is calculated from the path analysis (Fig. 3) by adding together the two paths from ethnicity to cord-blood leptin level: one that goes from ethnicity through fasting glucose level to cord-blood leptin level (0.18 × 0.26 = 0.047) and one that goes from ethnicity to fasting glucose level, and from fasting glucose level to cord-blood insulin level and from there to cord-blood leptin level (0.18 × 0.19 × 0.31 = 0.011). The path analysis shows that the difference in cord-blood leptin level between Pakistani and white British infants was completely explained by higher maternal fasting glucose levels amongst Pakistani women, acting both directly on cord-blood leptin levels and explaining 0.047 SD (81%) of the difference and also acting via cord-blood insulin levels, with that pathway explaining 0.019 SD (19%) of the difference.

## Discussion

We found positive linear associations of gestational fasting and postload glucose levels with cord-blood levels of insulin and leptin in both Pakistani and white British women. Pakistani infants had higher cord-blood leptin levels, indicating greater fat mass at birth, compared with white British infants. The magnitude of this difference was modest, with Pakistani infants having 16% higher levels of cord-blood leptin, equating to a 0.06SD difference. Given that the birthweight of Pakistani infants was >250 g lower than that of white British infants, this difference highlights a very different body composition between the two ethnic groups. We found that this greater birth adiposity was largely explained by higher gestational fasting glucose levels in women of Pakistani origin compared with white British women, which appears to act both directly on cord-blood leptin levels and also via increased fetal insulin secretion (as measured here by cord-blood insulin levels).

Glucose freely crosses the placenta; therefore, the amount delivered to the developing fetus depends upon maternal circulating levels [15–17]. Glucose levels are higher in Pakistani than white British women, thus the developing fetuses of Pakistani women will be relatively overnourished with glucose, which will stimulate increased fetal pancreatic secretion of insulin, which is a growth and fat deposition hormone. Our regression and path analysis results support these pathways and our original hypothesis that higher cord-blood leptin levels (birth adiposity) in Pakistani infants is driven by higher circulating gestational glucose levels in Pakistani women.

We undertook analyses both including and excluding women who met clinical criteria for GDM. In the Bradford Royal Infirmary, once identified, women with GDM will have been treated as clinically indicated with diet and exercise, metformin, or insulin to achieve and maintain euglycaemia. Treatment protocols are applied identically to women of all ethnicities. Women identified as having GDM are likely to have had lower glucose levels on average during the last trimester than if they had not been identified and treated; however, these women represent the upper end of the risk distribution and will have had higher glucose levels in earlier pregnancy and we felt it was important to examine relationships in the cohort with these women included as well as with them excluded. Overall, the mediation analyses were similar in cohorts that included or excluded women with GDM, with some minor differences between each stage of the regression and path analyses. GDM was more common in Pakistani than white British women, consistent with their higher levels of fasting and postload glucose, but in both ethnic groups most women were diagnosed with GDM on the basis of having elevated postload glucose levels. However, fasting glucose level was more strongly related to cord-blood levels of insulin and leptin than was postload glucose level, thus irrespective of inclusion or exclusion of those with GDM, the mediation by fasting glucose levels was similar.

To the best of our knowledge, this study is the first to examine the possibility that birth differences in cord-blood leptin levels between South Asian and white European populations is mediated by differences in gestational glycaemia. We used cord-blood insulin levels as a proxy for fetal insulin secretion and cord-blood leptin levels as a proxy for birth fatness. Maternal insulin does not cross the placenta and the fetus secrets insulin in relation to maternal levels of circulating glucose; the anabolic effects of insulin are then key for fetal fat deposition [15–17]. However, cord-blood insulin levels may also reflect insulin sensitivity at birth. Whilst it is difficult to determine how much of the variation in cord-blood insulin levels was due to offspring insulin sensitivity at birth, this sensitivity is likely to reflect only a small proportion of the variation, with variation largely reflecting fetal secretion [35, 36]. Leptin is found in fetal adipose tissue, and cord-blood leptin level is strongly positively related to fetal fat mass, explaining approximately 20–25% of variation in fat mass [26, 27], similar to the variation of birthweight that we found here. Whilst it is known that the placenta secretes leptin, the vast majority of this leptin is secreted into the maternal circulation, with less than 1% of cord-blood leptin estimated to be from the placenta/maternal circulation [37, 38]. Recent findings show that maternal gestational serum leptin level is not associated with cord-blood leptin level and is higher in women with a fetus with intrauterine growth restriction, whereas cord-blood leptin level is positively associated with birthweight [38]. These findings support cord-blood leptin level as reflecting fat mass at birth. Whilst we are unaware of any study examining whether cord-blood leptin level relates to fat mass with the same magnitude in European and South Asian infants, we found that the association of cord-blood leptin level with birthweight was the same in both groups, which is consistent with our previous more detailed study of these relationships [39].

Our study is observational and cannot prove causality. To assume the observed associations represent causal paths means assuming that measurement error between ethnicity and the mediators, and the mediators and cord-blood leptin, are minimal and/or are not correlated with each other [40]. Ethnicity was self-reported but verified by family tree information and country of birth of family members. Gestational glucose and cord-blood measurements were completed using valid procedures and with the laboratory staff unaware of participant characteristics. Claiming causal mediation would also assume that there is no residual confounding. Mendelian randomisation (using genetic variants that are known to be robustly associated with fasting glucose levels as proxies for its causal effect) has shown a causal effect of fasting glucose levels on birthweight, which together with the known physiology of pregnancy in relation to maternal glucose and fetal insulin, supports a causal effect of fasting glucose levels on fetal fatness. However, further replication of these findings and testing causal pathways through randomised controlled trials and/or Mendelian randomisation is warranted.

The South Asian participants in our study are all of Pakistani origin and we cannot assume that our results necessarily generalise to other South Asian populations. Likewise, the white British population may not be representative of all white European populations. However, other studies suggest similar differences in infant fat mass between Indian and white European infants [7–9].

Leptin regulates energy homeostasis by relaying information about the body’s energy and nutrient stores from the periphery to the brain [41]. However, adult humans in Western populations are largely resistant to this regulatory mechanism, with recent evidence suggesting that leptin resistance is present in children as young as 7 and 12 years of age [42, 43]. By contrast, two studies of 200 and 600 participants have shown that cord-blood leptin levels are inversely related to BMI at age 2–3 years [44, 45], with these findings interpreted as showing that there might be a period of leptin sensitivity in early human life. Both of these studies were conducted in European origin children and whether similar results would be seen in South Asian infants is yet to be determined. Furthermore, the substantial evidence of greater fatness, insulin resistance, type 2 diabetes and markedly higher leptin levels in South Asian children and adults compared with Europeans [1, 11–14, 20–25] argues against any leptin sensitivity in infancy having a lasting beneficial impact, but rather that higher leptin and fat mass levels are maintained in South Asians.

Our results provide further evidence of the higher prevalence of hyperglycaemia in South Asian than white British women and the potential adverse effects in terms of greater adiposity in the offspring of South Asian women. These findings suggest that current guidelines for the detection and control of hyperglycaemia and GDM in South Asian women should be rigorously applied. Selecting women for GDM testing on the basis of risk factors, as currently practised in most of the UK and many other countries, could miss nearly half of all cases of GDM in South Asian women because of failure to rigorously apply risk factor screening [46, 47]. Furthermore, to our knowledge, there is no randomised controlled trial evidence of the effect of treating GDM (or those at risk of GDM) specifically in South Asian women, despite their greater risk and differing lifestyles compared with white European women. In the most recent Cochrane systematic review, which included eight trials, none seem to have included any or sufficient South Asian women to determine effects in that population [48]. We were able to identify just one trial in South Asian women, which was a pilot randomised trial in India that aimed to compare the relative effects on GDM of two different types of insulin therapy [49].

In conclusion, we have shown that compared with white British infants, Pakistani infants have higher absolute cord-blood leptin levels, reflecting greater birth fat mass despite their lower birthweight. Importantly, we found that higher maternal gestational glucose levels explain this greater birth fat mass. Given the rapidly rising levels of diabetes in South Asian populations, these results warrant further exploration and, if replicated, the possibility of more aggressive identification and treatment of hyperglycaemia in pregnancy in South Asian women should be explored.

## Electronic supplementary material

Below is the link to the electronic supplementary material.

ESM Methods (PDF 445 kb)

ESM Fig. 1 (PDF 286 kb)

ESM Fig. 2 (PDF 477 kb)

ESM Fig. 3 (PDF 420 kb)

ESM Fig. 4 (PDF 238 kb)

ESM Table 1 (PDF 211 kb)

ESM Table 2 (PDF 222 kb)

ESM Table 3 (PDF 268 kb)

ESM Table 4 (PDF 268 kb)

## Abbreviations

BiB: Born in Bradford
GDM: Gestational diabetes mellitus
