# Supplementary material for: Pregnancy glycaemia and cord-blood levels of insulin and leptin in Pakistani and white British mother–offspring pairs: findings from a prospective pregnancy cohort
Source: Diabetologia. 2014 Oct 3;57(12):2492–500. doi: 10.1007/s00125-014-3386-6 (PMC4218974; doi:10.1007/s00125-014-3386-6)
Supplement: Supplementary file 1 — (PDF 445 kb) [file 125_2014_3386_MOESM1_ESM.pdf]

Supplementary methodological detail

1. ***<u>Questions on ethnic groups</u>***

We used UK Office of National Statistics (ONS) ethnic group categories:

Women were asked at interview to choose one option that best described their ethnic group or background from the ONS list, which is detailed below:

White

1. English / Welsh / Scottish / Northern Irish / British

2. Irish

3. Gypsy or Irish Traveller

4. Any other White background, please describe

Mixed / Multiple ethnic groups

5. White and Black Caribbean

6. White and Black African

7. White and Asian

8. Any other Mixed / Multiple ethnic background, please describe

Asian / Asian British

9. Indian

10. Pakistani

11. Bangladeshi

12. Chinese

13. Any other Asian background, please describe

Black / African / Caribbean / Black British

14. African

15. Caribbean

16. Any other Black / African / Caribbean background, please describe

Other ethnic group

17. Arab

18. Any other ethnic group, please describe

We compared Pakistani participants (those who indicated that they were in category 9) to White British participants (those who indicated that they were in category 1), both highlighted with bold italics above.

2. ***Derivation of UK equivalised maternal educational attainment***

We collected mothers highest educational qualification obtained; including qualifications obtained in the UK, Pakistan and elsewhere overseas in the baseline questionnaire. The first question lists UK qualifications using 11 qualification categories, “overseas”, “no qualifications” and “don’t know” categories. For qualifications obtained in Pakistan there are 10 different qualification categories, “no qualifications” and “don’t know” categories. For qualifications obtained elsewhere free text fields were used to record the qualification and country the qualification was obtained in.

For this study participants either reported UK or Pakistani qualifications, or that they had no qualifications or did not know; there were no participants with qualifications from a different country. The UK National Academic Recognition Information Center (UK NARIC; http://www.ecctis.co.uk/naric/Default.aspx) look-up tables were used to convert Pakistani qualifications to UK equivalents. Below we show how the qualifications from the UK and Pakistan map to the re-coded education categories used in this study.
NB: Don’t know relates to the mother responding “don’t know” during interview (this also includes verbal / free text responses of “Don’t know” and “Unknown”).

**Collapsed 5-mutually exclusive categories used in analyses**	**UK qualifications**	**Pakistani qualifications**
<5 GCSE equivalent	No qualifications<br><br>1 + 0 levels/CSEs/GCEs(any grades)<br>NVQ Level 1, Foundation GNVQ	No qualifications
5+ GCSE equivalent	5 + 0 levels, 5+ CSEs (grade 1) 5 + GCSEs, School Certificate<br>NVQ Level 2,Intermediate GNVQ	Second School Certificate (SSC) Matriculation
A-level equivalent	1 + A levels/AS levels<br>2 + A levels, 4 + AS levels, Higher School certificate<br>NVQ Level 3, Advanced GNVQ	Diploma in Commerce<br>Higher Secondary (HSC) Cert/Intermediate humanities, pre-eng, pre-med
Higher	NVQ Levels 4-5, HNC, HND<br>First degree (e.g. BA, BSc)<br>Post-graduate qualification (e.g. Post-graduate diploma, MA, MSc, PhD)	Certificate or Diploma from Board of technical education<br>Final Apprenticeship Certificate/Grade 2 skilled<br>Vocational institute diploma/grade 3 skilled worker certificate<br>Bachelor Degree (4 year) in generally professional fields<br>Bachelor Arts/ Commerce/ Engineering/ Science/ Technology (Pass or Honours)<br>Post-graduate qualification (e.g. MA, MSc, PhD)
Other	Other qualifications (e.g. City and Guilds, RSA/OCR, BTEC)<br>Don’t know	Don’t know

UK school and college qualifications have the following features (University/higher education qualifications are internationally recognised and similar to the equivalent from any other country):

O-levels: Ordinary-level (qualifications usually take at 15 (minimum legal school leaving age in the UK until 1st September 1972) or 16 years (minimum legal school leaving age in the UK from xx to present date) by students considered to have a high academic ability. Students would commonly sit between 5-10 O-levels in different subjects. These qualifications were replaced by the GCSE in 1986.

CSE: Certificate of Secondary Education. Qualifications usually take at 15 (minimum legal school leaving age in the UK until 1st September 1972) or 16 years (minimum legal school leaving age in the UK from 1972 to present date) by students considered to have a lower academic ability not suited to the standards required for O-level. Students would commonly sit between 5-10 CSE in different subjects. Student could take a mixture of CSE and O-levels in different subjects. For the purpose of categorising participants those who had both O-levels and CSE, but no other qualifications, would have their O-levels taken as their highest level of qualification. These qualifications were replaced by the GCSE in 1986.

GCSE: General Certificate of Secondary Education. Qualifications usually taken at 16 years (legal minimum school leaving age in the UK). This single qualification with a wider grading range replaced the O-level and CSE in 1986. Students would commonly sit 5-10 GCSE.

AS-level: Advanced subsidiary level: Qualifications usually taken in the first year of A-level studies and intermediate between O- and A-level. Commonly take in 5-6 subjects and used to decide which to focus on for A-levels.

3. ***Selection of covariables***

We selected likely confounding variables and other covariables *a priori* based on established associations and using directed acyclic graphs (DAGs) to identify any potential for introducing selection or other sources of bias.[1] Few characteristics could plausibly influence a woman's ethnicity and therefore confound the association of this with our outcomes (cord-blood leptin, cord-blood insulin and birth fat-free mass).[1,2] However, since we were interested in mediation pathways between maternal measures of glycaemia and the birth outcomes, and whether these might be causal, it was important to adjust for potential confounders between those mediators and outcomes. In the mediation analyses it would also be important to adjust for those potential confounders in the association of maternal ethnic origin and birth outcomes to avoid collider bias introducing confounding between ethnicity and the outcomes; from the DAG we drew it was clear that ethnicity and confounders are "*common causes*" of maternal glucose (the mediator), thus conditioning on maternal glucose would open a pathway linking the confounders to ethnicity and introducing confounding between ethnicity and cord-blood leptin if we did not adjust for these characteristics.[3]

The confounders that we adjusted for in this regard were maternal age, parity, BMI, smoking and socioeconomic position all of which are known to be associated with pregnancy glucose levels / gestational diabetes (GDM) risk[4-6] and also with birth size, and with cord-leptin levels in studies that have examined that.[7-10] Maternal education was used as our measure of socioeconomic position because we were able to equivalise this in both ethnic groups (see below), whereas for example few Pakistani women worked and so could not be allocated occupational social class and there were higher levels of missing data / do not know response for questions related to household income than for education. Maternal education differed between the two ethic groups and was related to gestational glucose and birth outcomes, often

in non-linear ways, and so this was included as a categorical variable (4 indicator variables) in all regression (including the path) analyses.

In addition to these confounding factors we also adjusted for infant sex, which could not influence ethnicity or maternal glucose status in pregnancy and so would not be a confounder, but is strongly associated with birth size and therefore adjusting for it could improve statistical efficiency. We ensured that associations did not differ by infant sex prior to this adjustment using a test for statistical interaction and also checked that adjusting for sex did not alter the point estimates of any associations. None of the associations examined in this study differed by offspring sex (all $p_{interaction} \geq 0.2$) and all regression analyses are presented with both sexes combined.

It is plausible that gestational glucose – particularly at high levels and where it reaches criteria for GDM – will be related to gestational age (because of induction of labour or elective early instrumental delivery), which in turn is related to birth size/fatness. In theory gestational age could mask the true relationship between maternal gestational glucose and cord-leptin (indicating birth fat mass), since those who are delivered early might not gain as much fat or skeletal growth as anticipated from the level of their maternal glucose and fetal insulin secretion response. In the main analyses we have therefore adjusted for gestational age. However, we also repeated all analyses without this adjustment and none of the associations differed when gestational age was not included in the models.

Lastly, we did not adjust for birthweight in our analyses. Other studies, including our own previous publications from the BiB cohort have done so, in order to examine whether birth fatness relative to a given birthweight is greater in South Asian compared with White

British.[10-13] However, here we were interested in absolute (not relative to birthweight) differences in birth fat mass as indicated by cord-blood leptin. Also since we know from previous analyses in the BiB cohort that (a) birthweight is lower in Pakistani compared to White British infants;[13] (b) cord-blood leptin is higher in Pakistani compared to White British infants[13] and (c) the magnitude of the correlation between birthweight and cord-blood leptin is the same in each ethnic group,[14] to adjust for birthweight in the ethnic difference in cord-blood leptin would statistically have to increase the magnitude of the difference and just what this means biologically is unclear.

References

1. Hernan MA, Hernandez-Diaz S, Werler MM, Mitchell AA. Causal knowledge as a prerequisite for confounding evaluation: an application to birth defects epidemiology. Am J Epidemiol. 2002;155:176-184.
2. Kawachi I, Daniels N, Robinson DE. Health disparities by race and class: why both matter. Health affairs. 2005;24:343-352.
3. Cole SR, Platt RW, Schisterman EF, Chu H, Westreich D, Richardson D, et al. Illustrating bias due to conditioning on a collider. Int J Epidemiol. 2010;39:417-420.
4. Solomon CG, Willett WC, Carey VJ, Rich-Edwards J, Hunter DJ, Colditz GA, et al. A prospective study of pregravid determinants of gestational diabetes mellitus. JAMA. 1997;278:1078-1083.
5. Dabelea D, Snell-Bergeon JK, Hartsfield CL, Bischoff KJ, Hamman RF, McDuffie RS. Increasing prevalence of gestational diabetes mellitus (GDM) over time and by birth cohort: Kaiser Permanente of Colorado GDM Screening Program. Diabetes Care. 2005;28:579-584.
6. Jovanovic L, Pettitt DJ. Gestational diabetes mellitus. JAMA. 2001;286:2516-2518.
7. Kramer MS. Determinants of low birth weight: methodological assessment and meta-analysis. Bull World Health Organ. 1987;65:663-737.
8. Davey Smith G, Leary S, Ness A, Lawlor DA. Challenges and novel approaches in the epidemiological study of early life influences on later disease. Adv Exp Med Biol. 2009;646:1-14.
9. West J. Explaining the differences in birth size and adiposity between Pakistani and white babies. PhD Thesis: University of Leeds; 2012.
10. Yajnik CS, Lubree HG, Rege SS, Naik SS, Deshpande JA, Deshpande SS, et al. Adiposity and hyperinsulinemia in Indians are present at birth. J Clin Endocrinol Metab. 2002;87:5575-5580.
11. Yajnik CS, Fall CH, Coyaji KJ, Hirve SS, Rao S, Barker DJ, et al. Neonatal anthropometry: the thin-fat Indian baby. The Pune Maternal Nutrition Study. Int J Obes Relat Metab Disord. 2003;27:173-180.

12. Krishnaveni GV, Hill JC, Veena SR, Leary SD, Saperia J, Chachyamma KJ, et al. Truncal adiposity is present at birth and in early childhood in South Indian children. Indian Pediatr. 2005;42:527-538.
13. West J, Lawlor DA, Fairley L, Bhopal R, Cameron N, McKinney PA, et al. UK-born Pakistani-origin infants are relatively more adipose than white British infants: findings from 8704 mother-offspring pairs in the Born-in-Bradford prospective birth cohort. J Epidemiol Community Health. 2013;67:544-551.
14. West JW, J; Fairley, L; Sattar, N; Whincup, P; Lawlor, D.A. Do ethnic differences in cord blood leptin levels differ by birthweight category? Findings from the Born in Bradford cohort study. International journal of Epidemiology. 2013;1–6. doi:10.1093/ije/dyt225.
