# Supplementary material for: Pregnancy glycaemia and cord-blood levels of insulin and leptin in Pakistani and white British mother–offspring pairs: findings from a prospective pregnancy cohort
Source: Diabetologia. 2014 Oct 3;57(12):2492–500. doi: 10.1007/s00125-014-3386-6 (PMC4218974; doi:10.1007/s00125-014-3386-6)

eFigure 1: Association of birth weight with cord-blood leptin in White British (N = 629) and Pakistani (N = 786) infants.

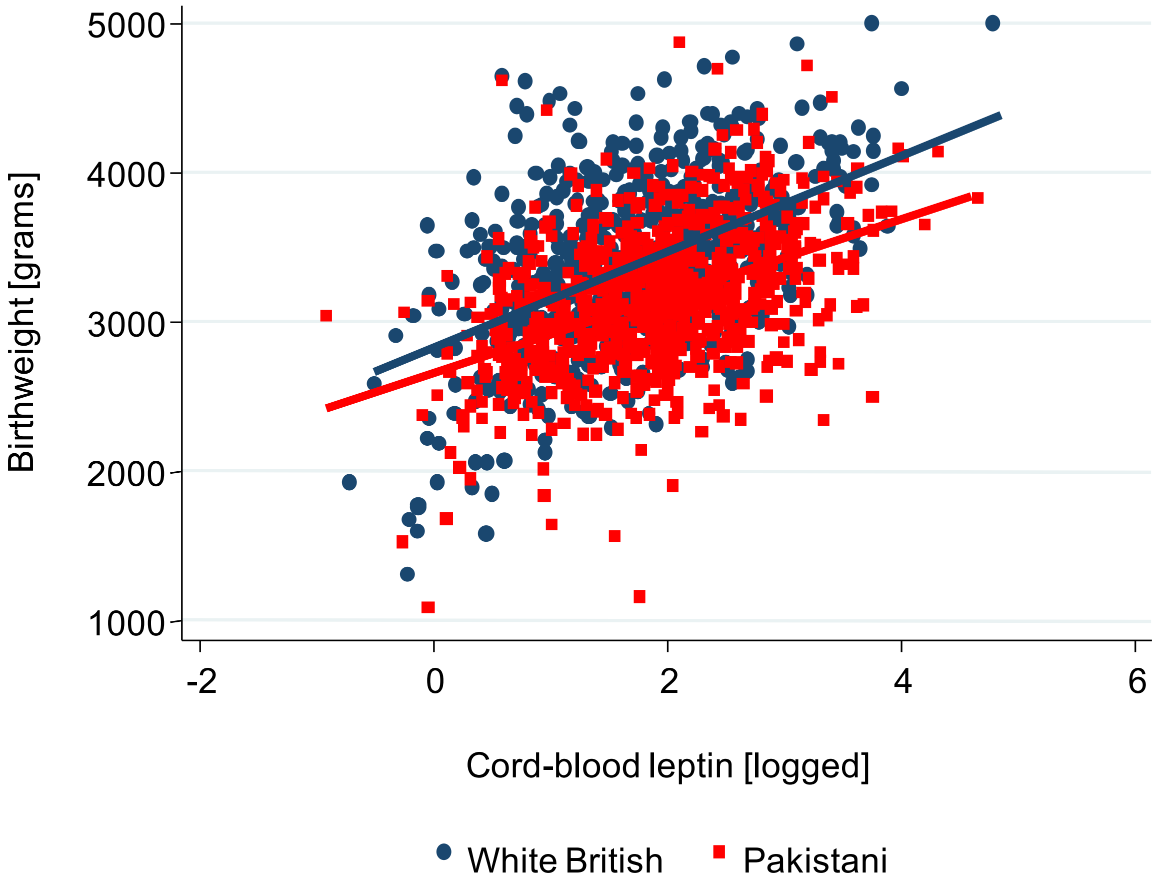

Supplement: Supplementary file 2 — (PDF 286 kb) [file 125_2014_3386_MOESM2_ESM.pdf]
