# Supplementary material for: Pregnancy glycaemia and cord-blood levels of insulin and leptin in Pakistani and white British mother–offspring pairs: findings from a prospective pregnancy cohort
Source: Diabetologia. 2014 Oct 3;57(12):2492–500. doi: 10.1007/s00125-014-3386-6 (PMC4218974; doi:10.1007/s00125-014-3386-6)

eFigure 2a: Association of maternal gestational postload glucose with infant cord-blood <u>insulin</u> in White British (N = 629) and Pakistani (N = 786) mother-infant pairs.

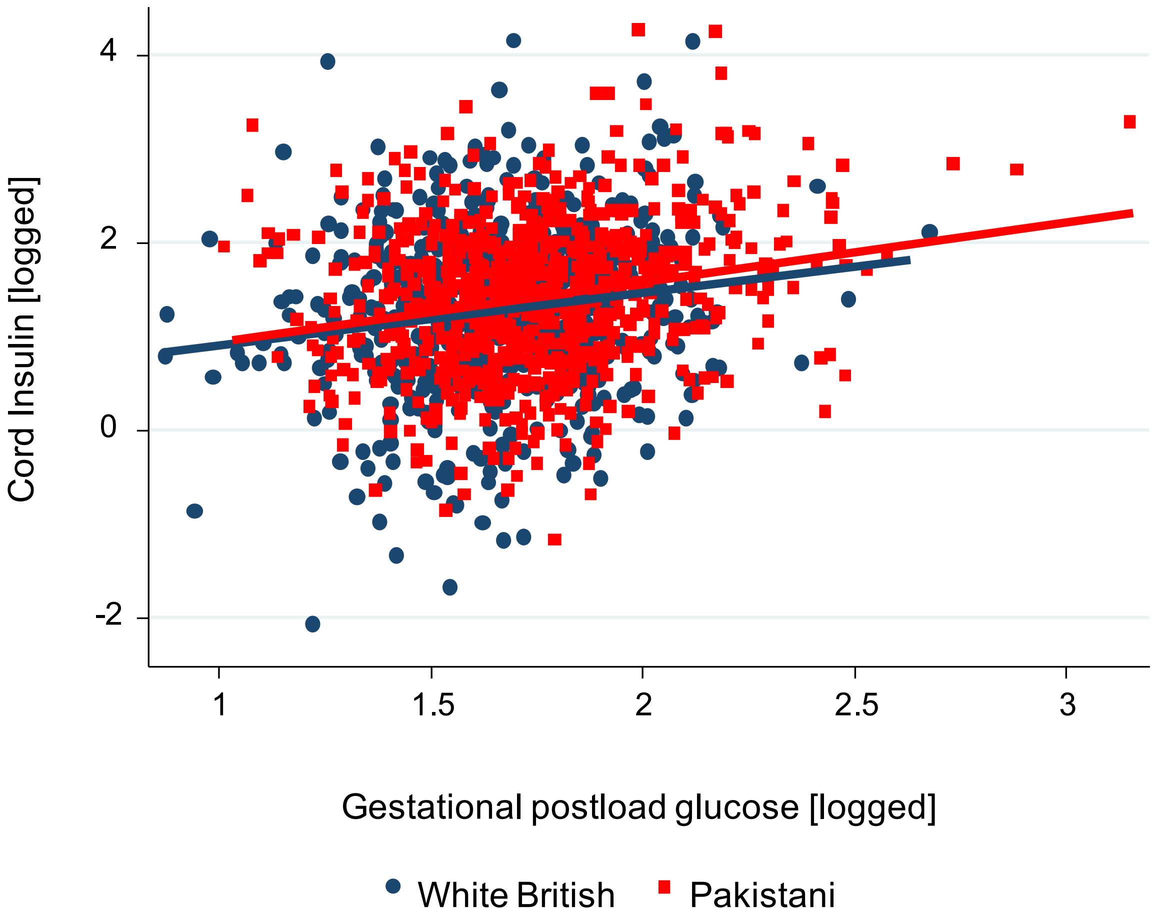

eFigure 2b: Association of maternal gestational postload glucose with infant cord-blood leptin in White British (N = 629) and Pakistani (N = 786) mother-infant pairs.

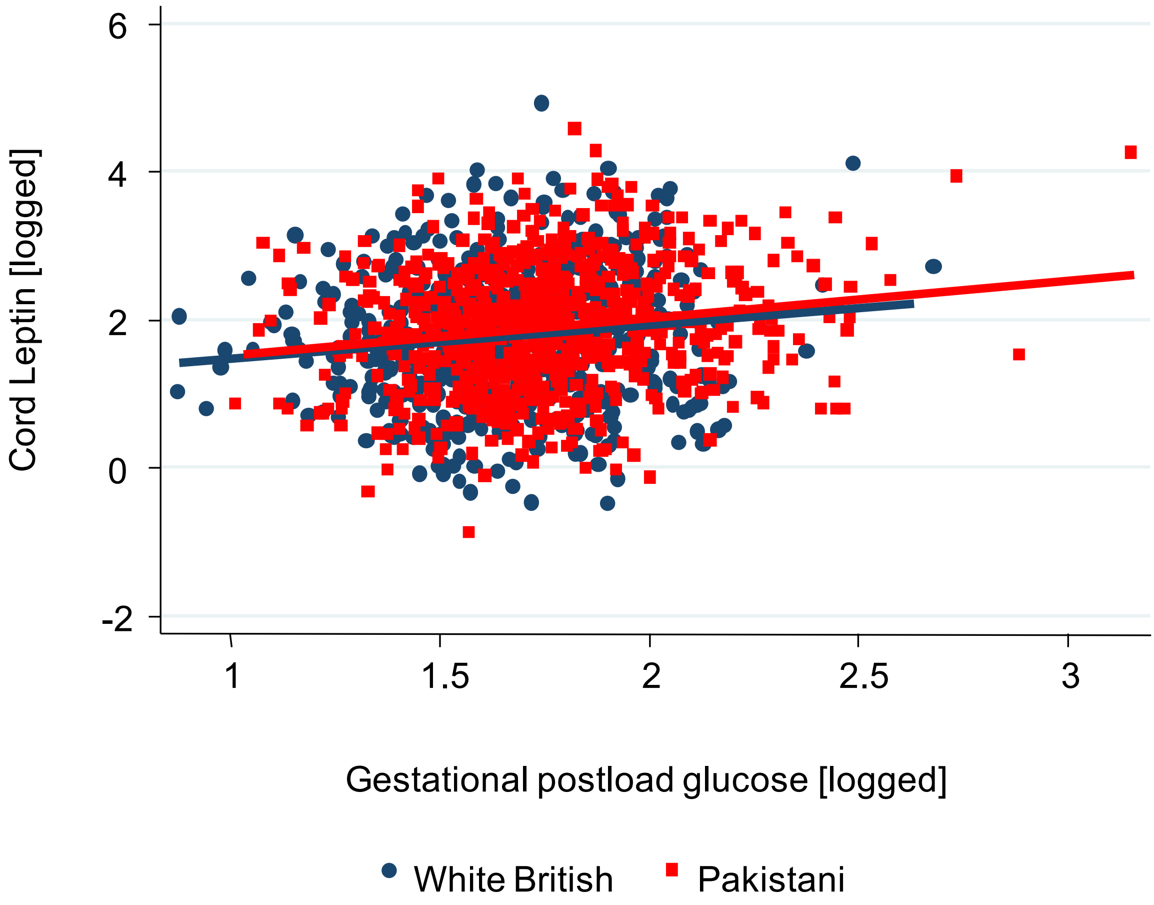

Supplement: Supplementary file 3 — (PDF 477 kb) [file 125_2014_3386_MOESM3_ESM.pdf]
