# Supplementary material for: Pregnancy glycaemia and cord-blood levels of insulin and leptin in Pakistani and white British mother–offspring pairs: findings from a prospective pregnancy cohort
Source: Diabetologia. 2014 Oct 3;57(12):2492–500. doi: 10.1007/s00125-014-3386-6 (PMC4218974; doi:10.1007/s00125-014-3386-6)

eFigure 3a: Association of maternal gestational fasting glucose with infant cord-blood <u>insulin</u> in White British (N = 596) and Pakistani (N = 689) mother-infant pairs in whom <u>mothers with gestational diabetes have been removed</u>.

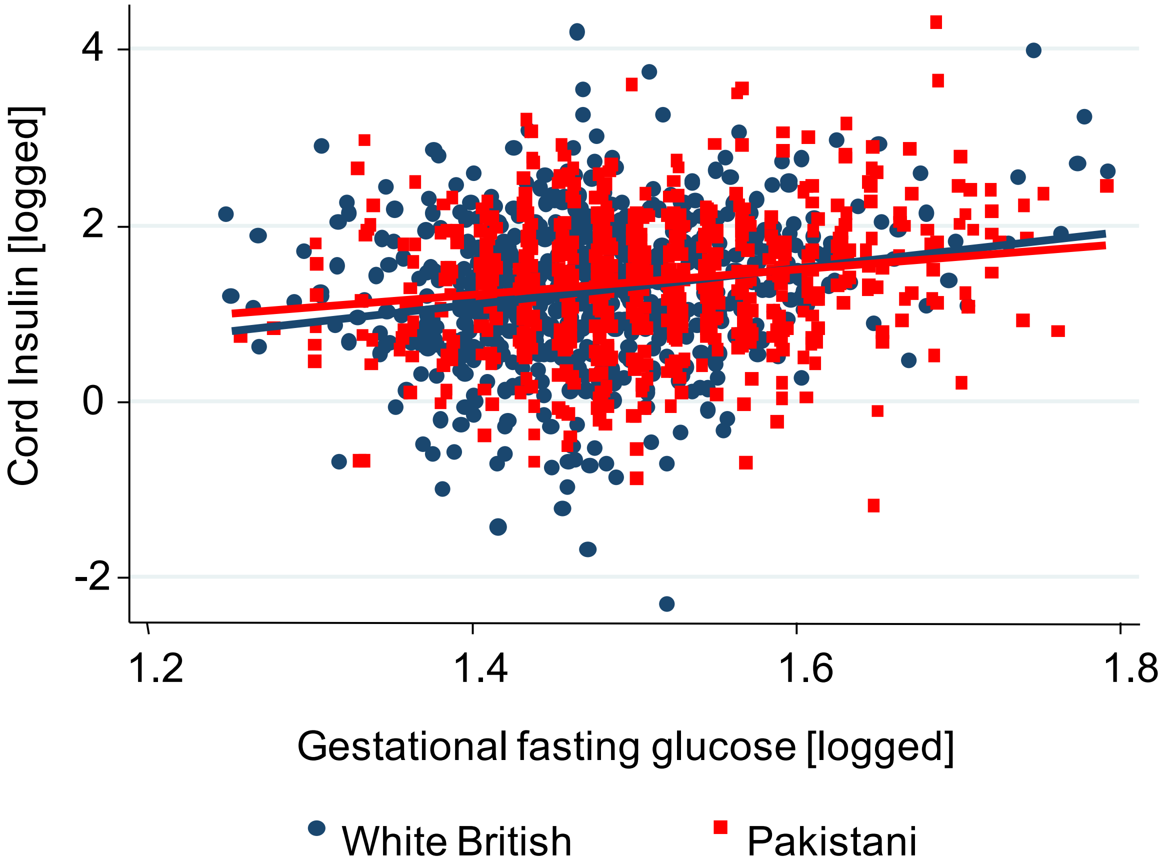

eFigure 3b: Association of maternal gestational fasting glucose with infant cord-blood leptin in White British (N = 596) and Pakistani (N = 689) mother-infant pairs in whom mothers with gestational diabetes have been removed.

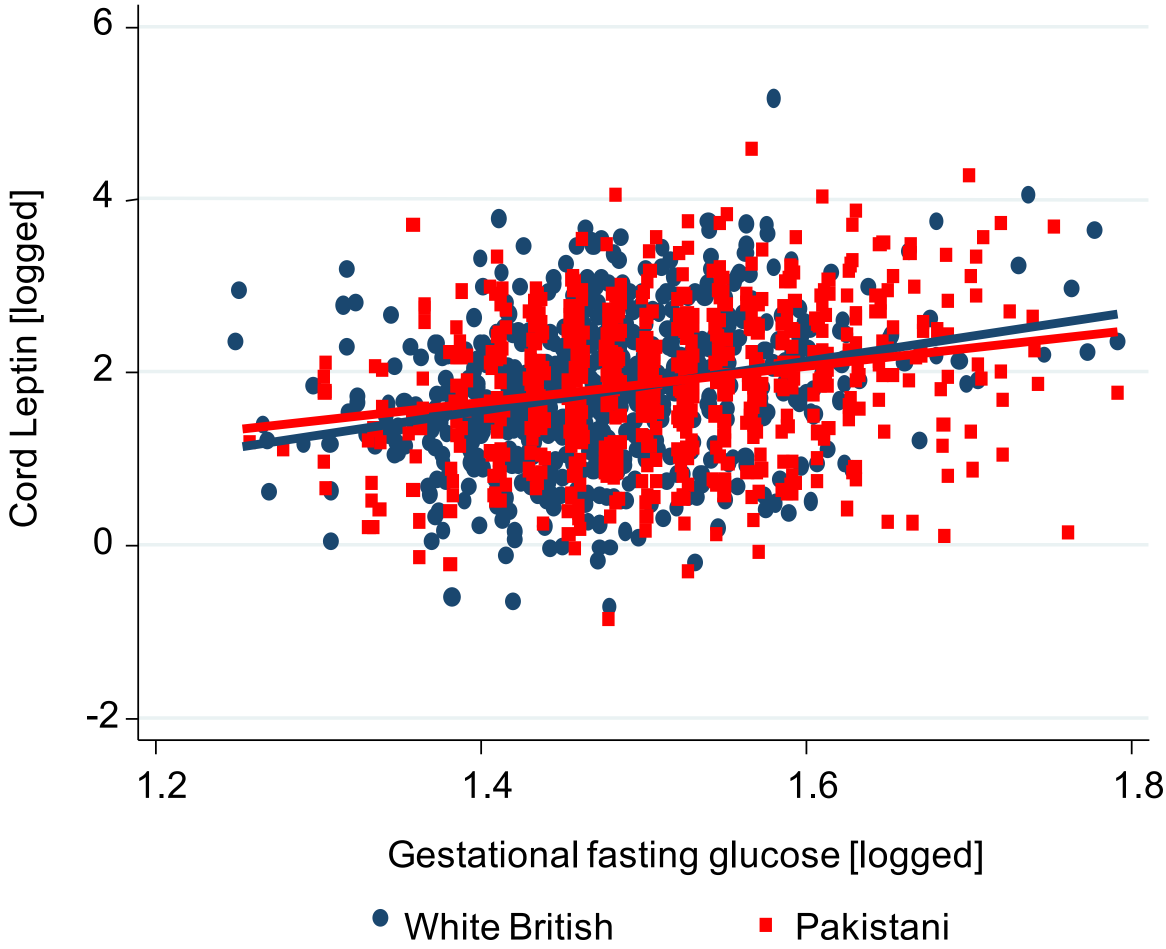

Supplement: Supplementary file 4 — (PDF 420 kb) [file 125_2014_3386_MOESM4_ESM.pdf]
