# Supplementary material for: Pregnancy glycaemia and cord-blood levels of insulin and leptin in Pakistani and white British mother–offspring pairs: findings from a prospective pregnancy cohort
Source: Diabetologia. 2014 Oct 3;57(12):2492–500. doi: 10.1007/s00125-014-3386-6 (PMC4218974; doi:10.1007/s00125-014-3386-6)
Supplement: Supplementary file 5 — (PDF 238 kb) [file 125_2014_3386_MOESM5_ESM.pdf]

eFigure 4: Path-analysis for differences in cord-leptin between Pakistani and White British individuals after removal of those with gestational diabetes. N = 1,285

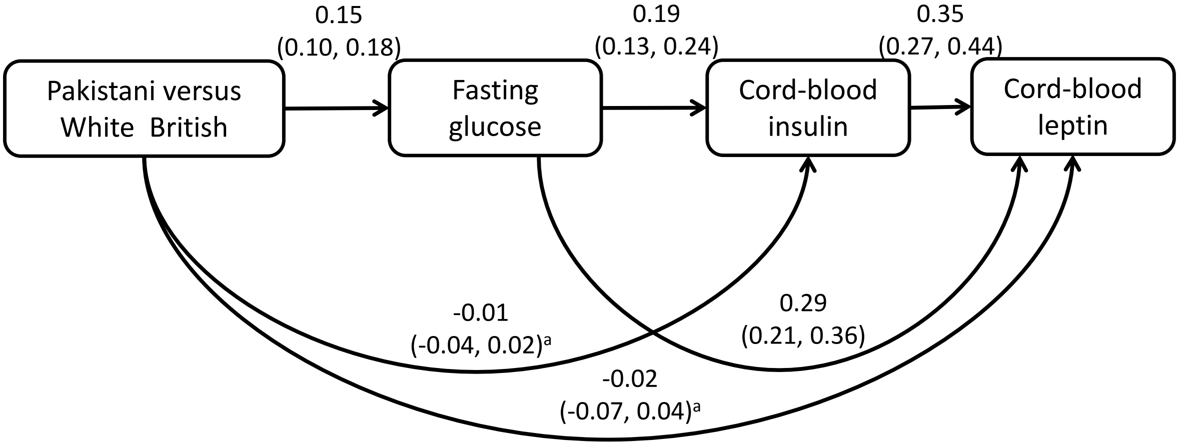

The numbers are all standardised regression coefficients, with their 95% confidence intervals in brackets, and are adjusted for maternal age, parity and education and infant sex and gestational age, in addition to other variables shown in the path analyses.
They are interpreted as the adjusted change in outcome (box at the end of the arrow head) in standard deviation units per category (for ethnic group) or per standard deviation of the exposure (arrow start).
P-values are $<$ 0.001 for all results except that indicated with [a] $p \geq 0.50$
