# Supplementary material for: Pregnancy glycaemia and cord-blood levels of insulin and leptin in Pakistani and white British mother–offspring pairs: findings from a prospective pregnancy cohort
Source: Diabetologia. 2014 Oct 3;57(12):2492–500. doi: 10.1007/s00125-014-3386-6 (PMC4218974; doi:10.1007/s00125-014-3386-6)
Supplement: Supplementary file 6 — (PDF 211 kb) [file 125_2014_3386_MOESM6_ESM.pdf]

eTable 1: Participant characteristics the subgroup included in this cord-blood study and in the whole BiB cohort (restricted to White British and Pakistani women only with singleton pregnancies and data on maternal gestational glucose).

**Characteristic**	**Unit / category**	**Median (IQR), means (SD) or N (%)[a]**	
		**Cord blood subgroup included in this study N = 1415**	**Whole BiB cohort[b] N = 8799**
Ethnicity	White British	629 (45)	4087 (46)
	Pakistani	786 (55)	4712 (54)
Age	Years	27.1 (5.7)	27.2 (5.6)
Fasting glucose	mmol/l	4.4 (4.2, 4.8)	4.4 (4.2, 4.7)
Postload glucose	mmol/l	5.5 (4.7, 6.4)	5.5 (4.7, 6.4)
BMI	Kg/m^2	26.2 (5.8)	26.2 (5.7)
Parity (number of previous pregnancies)	0	504 (36%)	3360 (40%)
	1	418 (30%)	2454 (29%)
	2	263 (19%)	1438 (17%)
	≥3	230 (16%)	1221 (14%)
Smoking	Never	985 (70%)	6014 (68%)
	Past	170 (12%)	1243 (14%)
	In pregnancy	260 (18%)	1528 (17%)
Education	<5GCSE	321 (23%)	2039 (23%)
	5 GCSE	467 (33%)	2872 (33%)
	A-level	210 (15%)	1280 (15%)
	Higher	327 (23%)	2001 (23%)
	Other	90 (6%)	590 (7%)
Gestational age	Weeks	39 (38, 40)	39 (38, 40)
Birthweight	Grams	3264 (525)	3248 (547)
Cord leptin	ng/ml	6.1 (3.4, 11.3)	NA
Cord insulin	pmol/l	27.8 (16.7, 47.2)	NA
Sex	Male	705 (50%)	4518 (51%)
	Female	710 (50%)	4281 (49%)

[a]For maternal age, maternal BMI and birthweight values are mean (SD), for maternal gestational fasting and postload glucose, cord-blood insulin and cord-blood leptin values are median (IQR); for all other variables (which are categorical) values are numbers (%).
[b] So that the two groups are a legitimate comparison we restricted the whole BiB cohort to those with the same inclusion criteria as our study, so these are all participants who are either White British or Pakistani with a singleton pregnancy who had data on gestational glucose.
IQR: Inter Quartile Range; SD: Standard Deviation; N: number; BMI: Body Mass Index; GCSE: General Certificate of Secondary Education; A-level: Advanced-level; NA: not applicable – the subgroup included in this study were defined by being in the cord blood sample study.
