# Supplementary material for: Pregnancy glycaemia and cord-blood levels of insulin and leptin in Pakistani and white British mother–offspring pairs: findings from a prospective pregnancy cohort
Source: Diabetologia. 2014 Oct 3;57(12):2492–500. doi: 10.1007/s00125-014-3386-6 (PMC4218974; doi:10.1007/s00125-014-3386-6)
Supplement: Supplementary file 7 — (PDF 222 kb) [file 125_2014_3386_MOESM7_ESM.pdf]

eTable 2: Participant characteristics by ethnicity and in the whole cohort. N = 1,415

Characteristic	Unit / category	Median (IQR), means (SD) or N (%)			$p_{ethnic\ differences}$[b]
		The whole cohort N = 1415	White British N = 629 (45%)	Pakistani N = 786 (55%)	
**Maternal characteristics**					
Age	Years	27.1 (5.7)	26.4 (6.1)	27.6 (5.1)	< 0.001
Fasting glucose[a]	mmol/l	4.4 (4.2, 4.8)	4.3 (4.2, 4.6)	4.5 (4.3, 4.9)	< 0.001
Postload glucose[a]	mmol/l	5.5 (4.7, 6.4)	5.3 (4.5, 6.2)	5.7 (4.9, 6.6)	< 0.001
BMI	Kg/m^2	26.2 (5.8)	27.1 (6.1)	25.4 (5.5)	< 0.001
Parity (number of previous pregnancies)	0	504 (36%)	277 (44%)	227 (29%)	< 0.001
	1	418 (30%)	201 (32%)	217 (28%)	
	2	263 (19%)	98 (16%)	165 (21%)	
	≥3	230 (16%)	53 (8%)	177 (23%)	
Smoking	Never	985 (70%)	255 (41%)	730 (93%)	< 0.001
	Past	170 (12%)	144 (23%)	26 (3%)	
	Pregnancy	260 (18%)	230 (36%)	30 (4%)	
Education	<5GCSE	321 (23%)	128 (20%)	193 (25%)	< 0.001
	5 GCSE	467 (33%)	220 (35%)	247 (31%)	
	A-level	210 (15%)	96 (15%)	114 (14%)	
	Higher	327 (23%)	127 (20%)	200 (25%)	
	Other	90 (6%)	58 (9%)	32 (4%)	
**Offspring characteristics**					
Gestational age[a]	weeks	39 (38, 40)	39 (38, 40)	39 (38, 40)	0.99
Birthweight	grams	3264 (525)	3400 (547)	3155 (479)	< 0.001
Cord-blood insulin[a]	pmol/l	27.8 (16.7, 47.2)	25.7 (15.3, 42.4)	29.2 (16.7, 48.0)	0.001
Cord-blood leptin[a]	ng/ml	6.1 (3.4, 11.3)	5.9 (3.2, 10.4)	6.9 (3.8, 12.0)	0.004
Sex	Male	705 (50%)	308 (49%)	397 (50)	0.56
	Female	710 (50%)	321 (51%)	389 (50)	

[a]For these continuously measured variables results are median (interquartile range; IQR), for all other continuously measured variables results are means (standard deviation; SD); categorical variables are given category labels in column two and results are numbers (%).
[b]p-values are from unpaired t-tests for all continuous variables (using logged values for blood based characteristics) and chi^2 tests for categorical variables. All are testing the null hypothesis that the distributions of characteristics do not differ between the two ethnic groups.
IQR: Inter Quartile Range; SD: Standard Deviation; N: number; BMI: Body Mass Index; GCSE: General Certificate of Secondary Education (qualifications normally taken at age 16 years, the minimum school leaving age in the UK); A-level: Advanced-level (qualifications usually take at age 18 and required for University and many professional training schemes).
