# Supplementary material for: Pregnancy glycaemia and cord-blood levels of insulin and leptin in Pakistani and white British mother–offspring pairs: findings from a prospective pregnancy cohort
Source: Diabetologia. 2014 Oct 3;57(12):2492–500. doi: 10.1007/s00125-014-3386-6 (PMC4218974; doi:10.1007/s00125-014-3386-6)
Supplement: Supplementary file 8 — (PDF 268 kb) [file 125_2014_3386_MOESM8_ESM.pdf]

eTable 3: Associations of gestational fasting and postload glucose with cord blood insulin leptin in white British and Pakistani women. Both unadjusted and confounder adjusted analyses in all participants including those with gestational diabetes. N = 1415

Outcome	Model[a]	Mean difference in outcome for each maternal exposure (95%CI)		
		White British N = 629	Pakistani N = 786	$P_{interaction}$[b]
**Exposure = Fasting glucose (per 1 mmol/l)**				
Cord insulin (%)	1: Unadjusted	38.2 (24.2, 52.0)	30.8 (22.6, 39.0)	0.36
	2: Confounder adjusted	30.2 (15.6, 44.8)	24.0 (15.4, 32.5)	0.43
Cord leptin (%)	1: Unadjusted	49.6 (35.9, 63.4)	30.5 (21.7, 39.3)	0.02
	2: Confounder adjusted	42.2 (29.0, 55.4)	29.4 (20.7, 38.0)	0.11
**Exposure = Postload (120 minutes) glucose (per 1mmol/l)**				
Cord insulin (%)	1: Unadjusted	9.7 (4.8, 14.5)	10.3 (7.4, 13.2)	0.83
	2: Confounder adjusted	6.9 (1.9, 11.2)	7.5 (4.5, 10.5)	0.91
Cord leptin (%)	1: Unadjusted	8.3 (3.4, 13.2)	7.5 (4.4, 10.7)	0.78
	2: Confounder adjusted	8.2 (3.6, 12.8)	7.8 (4.8, 10.9)	0.79
**Exposure = Cord-blood insulin (per 1pmol/l)**				
Cord leptin (%)	1: Unadjusted	0.5 (0.4, 0.6)	0.6 (0.5, 0.8)	0.16
	2: Confounder adjusted	0.6 (0.4, 0.7)	0.6 (0.5, 0.7)	0.55

All results are mean differences in the outcome expressed on a percentage scale; the null value is 0 for all results.

[a] Model 1: unadjusted

Model 2: adjusted for potential confounders: maternal age, BMI, parity, smoking, education and gestational age and offspring sex

[b] Testing the null hypothesis that associations differ between White British and Pakistani pairs

N = number; CI = Confidence interval
